# Dietary Supplementation With Magnolia Bark Extract Alters Chicken Intestinal Metabolite Levels

**DOI:** 10.3389/fvets.2020.00157

**Published:** 2020-03-24

**Authors:** Inkyung Park, Sungtaek Oh, Erik. P. Lillehoj, Hyun S. Lillehoj

**Affiliations:** ^1^Animal Bioscience and Biotechnology Laboratory, Beltsville Agricultural Research Center, Agricultural Research Service, United States Department of Agriculture, Beltsville, MD, United States; ^2^Neuroregeneration and Stem Cell Programs, Institute for Cell Engineering, Johns Hopkins University School of Medicine, Baltimore, MD, United States; ^3^Department of Pediatrics, University of Maryland School of Medicine, Baltimore, MD, United States

**Keywords:** phytochemical, intestine, metabolomics, growth, chicken, magnolia

## Abstract

Magnolia bark extract administered as a dietary supplement to poultry confers a performance and health benefit, but the mechanisms are unknown. Here, a metabolomics approach was used to identify changes in intestinal metabolite levels in chickens fed an unsupplemented diet or a diet supplemented with magnolia bark extract. Total body weight gains of chickens fed magnolia bark-supplemented diets were increased 2% (from 861 to 878 g/chicken), compared with chickens fed an unsupplemented diet. Compared with unsupplemented controls, the levels of 278 intestinal biochemicals (metabolites) were altered (165 increased, 113 decreased) in chickens given the magnolia-supplemented diet. Data for biochemicals of intestinal contents of chickens fed the unsupplemented diet clustered on the left side of the PCA score plot, while those of the magnolia-supplemented diet were separated and clustered on the right side. The biochemicals included changes in the levels of amino acids, fatty acids, peptides, and nucleosides, which provided a distinctive biochemical signature unique to the magnolia-supplemented group, compared with the unsupplemented group. These results provide the foundation for future studies to identify naturally-produced biochemicals that might be used to improve poultry growth performance.

## Introduction

Regulatory agencies warn that the rise of multidrug-resistant bacterial pathogens could potentially be the greatest threat to human health of our time (1, 2). Antibiotic-resistant bacteria are estimated to kill more than 100 million people worldwide per year by 2050. Among the causes for the development and spread of antibiotic-resistant human pathogens is the overuse of antibiotic growth promoters (AGPs) in food animal production. Animal agriculture is responsible for more than 70% of total antibiotic usage worldwide, principally as in-feed additives for growth promotion and prophylactic disease control (3). In commercial poultry production, AGPs have been used to increase the growth rate and improve feed conversion (kg body weight gain per kg feed) since the 1940's (4). While it is clear that dietary supplementation of chickens with antibiotics alters the intestinal microbiome and increases the bioavailability of nutrients to the host, the exact mechanism of action of AGPs is unknown (5–7). As a result, formulating a rationale scientific approach to the identification of non-antibiotic alternatives that provide a growth enhancing effect without the potential for development of drug resistance has been impeded.

The magnolia plant contains a variety of chemical compounds with demonstrated bioactive properties, and both the bark and flower buds of *Magnolia officinalis* have been used for hundreds of years in traditional human medicine (8). Magnolia extracts and its purified components have been demonstrated to have anti-cancer effects, as well as for treating a variety of neuronal, inflammatory, cardiovascular, and gastrointestinal disorders (8–11). Few studies, however, have investigated the medicinal effects of magnolia in veterinary medicine (12). We previously reported that two commonly-used antibiotics, bacitracin and virginiamycin, alter the intestinal metabolome when given as dietary AGPs to broiler chickens (13). Therefore, the current study was performed to characterize the metabolic changes in the chicken intestine following dietary supplementation with an extract of *M. officinalis* bark in order to identify biochemical compounds that might serve as alternatives to AGPs for improving poultry growth performance.

## Materials and Methods

### Animals and Ethics Statement

One-day-old male Ross 708 commercial broiler chickens (Longenecker's Hatchery, Elizabethtown, PA) were randomly divided into two treatment groups (*n* = 16/group). The chickens were housed in starter cages from day 1 to day 14 of age prior to transfer to finisher cages where they were kept until sacrifice on day 21. All chickens were housed in the same room and provided *ad libitum* access to feed and water. Each cage was 0.65 m in width and 0.75 m in length (14 chickens/m^2^). The guidelines for the care and use of animals in agriculture research were followed throughout the entire experiment, and all animal protocols (# 16-001) were approved by the Institutional Animal Care and Use Committee of the Beltsville Agriculture Research Center.

### Preparation of Magnolia Bark Extract

An extract of *M. officinalis* bark (Pancosma, Geneva, Switzerland) was prepared as previously described (12). Briefly, the bark was washed, dried at 50°C to a dry matter content of 90%, and comminuted. The dried material was subjected to supercritical CO_2_ fluid extraction at 1200–1400 L/h for 3.5 h at 25–30 MPa and 35–40°C, and the extract taken up in ethanol.

### Experimental Design, Growth Performance, and Intestinal Metabolomics Analysis

Chickens were fed a corn-soybean meal-based diet formulated to meet the requirements for chickens as suggested by National Research Council (Table 1). Animals in the unsupplemented, control diet group were provided the basal diet alone, while those in the magnolia bark extract group were provided with the basal diet supplemented with 0.33 mg/kg of the magnolia extract. Feed additions were weighed and recorded daily, and feeders were shaken once per day. The chickens were weighed at 21 days of age for calculation of growth performance. At 21 days of age, 8 chickens from the unsupplemented control group and 8 chickens from magnolia-supplemented group were euthanized by cervical dislocation and the intestine harvested. Intestinal contents were collected aseptically by gently finger-stripping the ileal segment, immediately placed on dry ice, and stored at −80°C. Global metabolomic profiling of the intestinal contents was performed by mass spectrometry (MS) (Metabolon, Durham, NC) as described (13). Raw data was extracted and processed using the DiscoveryHD4 global metabolomics platform. Compounds were identified by comparison to library entries of purified standards or recurrent unknown entities based on retention index, accurate mass match to the library within 10 ppm, and MS/MS forward and reverse scores between experimental data and authentic standards. MS/MS scores were based on comparison of the ions present in the experimental spectrum to the ions present in the library spectrum.

**Table 1:** Composition of the basal diet.

**Ingredient**	****%****
Corn	59.01
Soybean meal	33.99
Soybean oil	2.75
Dicalcium phosphate	2.00
Calcium carbonate	1.40
Salt	0.35
Poultry vitamin mix^*a*^	0.20
Poultry mineral mix^*b*^	0.15
DL-Methionine	0.10
Choline chloride (60%)	0.05
Total	100.0
**Calculated nutrient composition**	****%****
Crude protein	18.0
Crude fiber	3.21
Crude oil	8.48
Ash	5.41
Calcium carbonate	1.19
Available phosphorus	0.54
Lysine	1.00
Methionine	0.42
Cysteine + Methionine	0.65
Metabolizable energy, Mcal/kg	3.59

a *The vitamin mixture provided the following nutrients per kg of diet: vitamin A, 2,000 IU; vitamin D_3_, 22 IU; vitamin E, 16 mg; vitamin K, 0.1 mg; thiamin, 3.4 mg; riboflavin, 1.8 mg; vitamin B_6_, 6.4 mg; vitamin B_12_, 0.013 mg; biotin, 0.17 mg; pantothenic acid, 8.7 mg; folic acid, 0.8 mg; niacin, 23.8 mg*.

b *The mineral mixture provided the following nutrients per kg of diet: Fe, 0.4 mg; Zn, 0.2 mg; Mn, 0.18 mg; Co, 0.0013 mg; Cu, 0.021 mg; Se, 0.0002 mg*.

### Statistical Analysis

Statistical analysis was performed as previously described (13). Each chicken was considered the experimental unit. The type of experimental diet was considered the treatment factor. Data were analyzed using a mixed model methodology (PROC MIXED, SAS Institute, Cary NC). For growth performance, mean ± SEM values were calculated for final body weight at day 21. Differences between means were compared using the two-tailed Student's t test with *p* ≤ 0.05 considered significantly different. ANOVA was used to identify the biochemicals whose levels were significantly altered between the unsupplemented and magnolia-supplemented groups following median scaling, log transformation, and imputation of missing values, if any, with the minimum value observed for each compound. All data were analyzed for outliers and one sample from the control group was excluded in the biochemical analysis. Standard statistical analyses of log-transformed data were performed using Array Studio software (OmicSoft, Cary, NC). For analyses that were not standard in Array Studio, the programs R (R Foundation for Statistical Computing, Vienna, Austria) or JMP (SAS Institute) were used. An estimate of the false discovery rate was obtained by calculating the q-value to account for the false positives that normally occur in metabolomics-based studies. Principle component analysis (PCA) was measured, which is a commonly used algorithm to analyze the metabolic profiling originating from different samples (14). Random forest analysis (RFA) was performed to identify metabolite signatures and the biochemical importance of the 30 most significantly altered metabolites for distinguishing the control vs. magnolia-supplemented groups.

## Results

### Dietary Magnolia Bark Extract Supplementation Increases Chicken Body Weight Gain

No differences in initial body weights between the two dietary groups at day 1 were observed (data not shown). Final body weights (878 ± 5.8 g) at day 21 of chickens fed the magnolia-supplemented diet were greater (*p* < 0.05) than those (861 ± 5.8 g) of chickens fed the unsupplemented diet, confirming the previously reported growth enhancing effect of chickens fed a diet supplemented with 0.33 mg the magnolia extract/kg (12).

### Dietary Magnolia Bark Extract Supplementation Affects Principle Component Analysis

**Figure 1:**
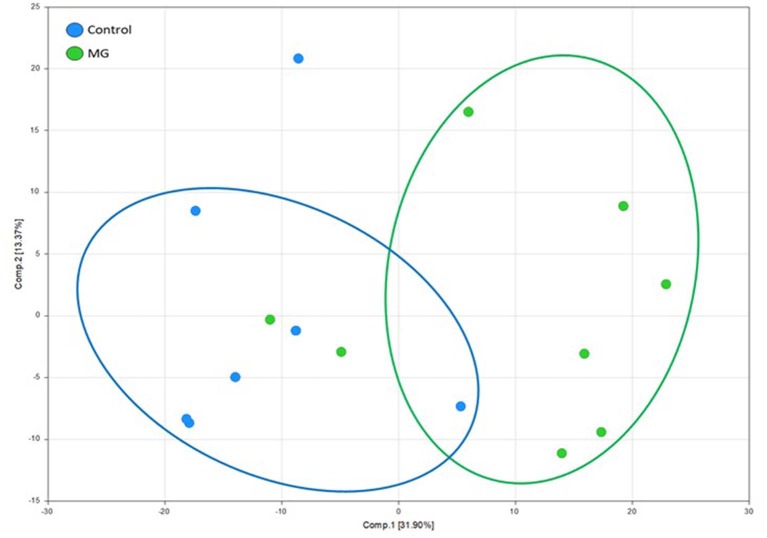
Principle component analysis score plot of identified metabolites in intestinal contents of chicken fed an unsupplemented, control diet (blue) or a diet supplemented with magnolia (MG) bark extract (green). Each dot on the plot represents an individual sample.

PCA revealed a district separation of identified intestinal metablolites between chickens fed the unsupplemented, control diet and those fed the magnolia-supplemented diet (Figure 1). *S* values for components 1 and 2 were 31.90 and 13.37%, respectively. Data for metabolites of intestinal contents of chickens fed the unsupplemented diet clustered on the left side of the PCA score plot, while those of the magnolia-supplemented diet were separated and clustered on the right side, indicating that feeding chickens with the magnolia bark extract could induce significant changes in the intestinal metabolomic profile, compared with feeding the unsupplemented diet.

### Dietary Magnolia Bark Extract Supplementation Alters Intestinal Metabolite Levels

A total of 706 biochemicals were identified in the intestinal contents of chickens fed an unsupplemented, control diet or a diet supplemented with magnolia bark extract. Of these, the levels of 423 metabolites were increased, 278 were decreased, and 5 were unchanged in the magnolia-supplemented vs. control groups. Of the 423 increased biochemicals, 165 were significantly increased, and of the 278 decreased metabolites, 113 were statistically significant (*p* < 0.05). Of 165 up-regulated biochemicals, 62 biochemicals and 57 biochemicals were related to lipid and amino acid metabolism, respectively. Otherwise, 19, 16, and 27 biochemicals of 113 down-regulated biochemicals were identified as metabolites related to amino acids, carbohydrates, and lipids, respectively.

### Intestinal Metabolite Signatures and Biochemical Importance Analyses

**Figure 2:**
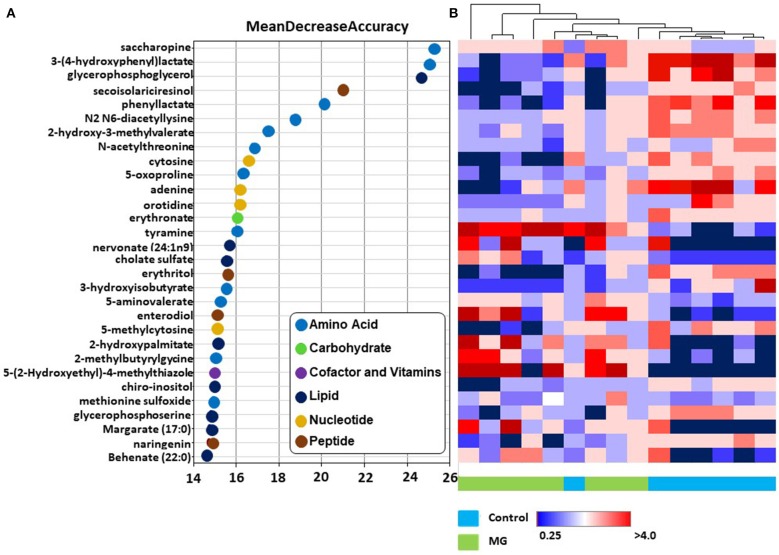
**(A)** Random forest plots of the top 30 biochemicals whose levels were altered in chickens fed the magnolia bark extract, compared with unsupplemented controls. Biochemicals are listed from bottom to top in increasing order of importance for contributing to the biochemical signatures separating the two treatment groups, and are plotted in color-coded symbols according to chemical classification. **(B)** Heat map showing hierarchical clustering using Ward's algorithm for the top 30 biochemicals identified by random forest analysis. The clustering along the abscissa (x-axis) is by samples of intestinal contents of chickens fed the magnolia-supplemented diet (green) or unsupplemented diet (blue) and along the ordinate (y-axis) by specific biochemicals indicated in **(A)**.

In RFA, metabolites of amino acids (40.0%), lipids (26.7%), nucleosides (13.3%), peptides (13.3%), vitamins and cofactors (0.03%), and carbohydrates (0.03%) accounted for the majority of biochemicals classified as the most important for distinguishing between the two treatment groups (Figure 2A). RFA of the control *vs*. magnolia groups gave a predictive accuracy of 73.3%, suggesting that these metabolites are candidate biomarkers for distinguishing between the two groups. Of 8 magnolia group samples, 6 were predicted to belong to the magnolia group and 2 to the control group (Table 2). Of 7 control group samples, 5 were predicted to belong to the control group and 2 to the magnolia group. Hierarchical clustering showed two distinct clusters based on their abundance in the control and magnolia supplementation groups (Figure 2B).

**Table 2:** Random forest analysis of the altered biochemicals distinguishing between the control (*n* = 7) *vs*. magnola-supplemented (*n* = 8) dietary groups.

		**Predicted group**		**Class error**
		**Control**	**Magnolia**	
Actual group	Control	5	2	28.6%
	Magnolia	2	6	25.0%

*Predictive accuracy = 73.3%*.

### Specific Intestinal Metabolites Altered Following Dietary Magnolia Bark Extract Supplementation

**Figure 3:**
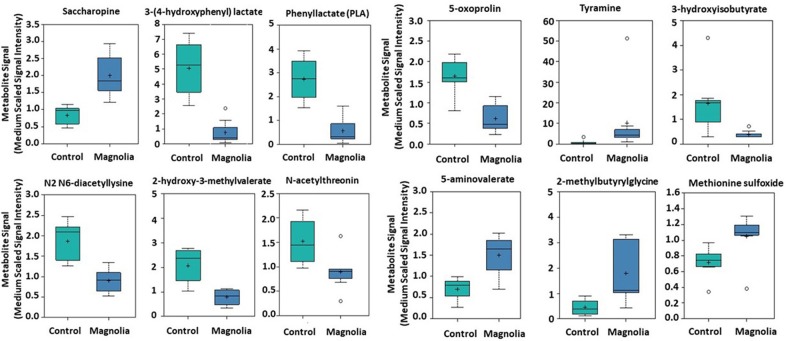
Box-and-whisker plots of the levels of amino acids in the intestine of chickens fed an unsupplemented, control diet (green) or a diet supplemented with magnolia bark extract (blue). The boxes represent the interquartile range (IQR) defined by the 25th and 75th percentiles. The horizontal line represents the median value. The cross represents the mean value. The upper whisker represents Q3 + (1.5 × IQR), while the lower whisker represents Q1 – (1.5 × IQR). Circles represent outliers.

**Figure 4:**
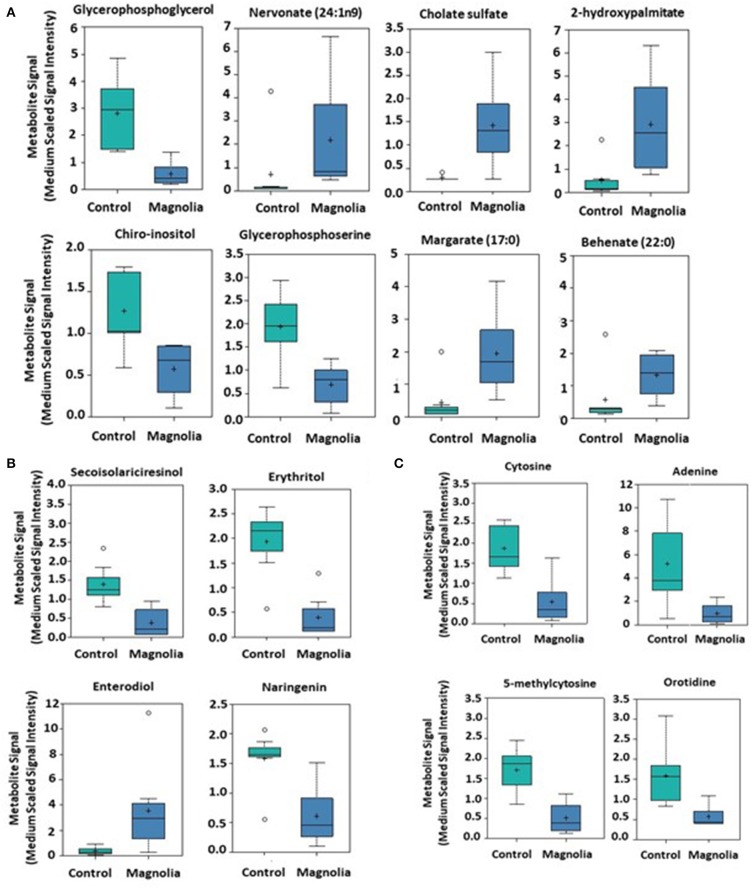
Box-and-whisker plots of the levels of **(A)** fatty acids, **(B)** peptides, and **(C)** nucleosides in the intestine of chickens fed an unsupplemented control diet (green) or a diet supplemented with magnolia bark extract (blue). The boxes represent the interquartile range (IQR) defined by the 25th and 75th percentiles. The horizontal line represents the median value. The cross represents the mean value. The upper whisker represents Q3 + (1.5 × IQR), while the lower whisker represents Q1 – (1.5 × IQR). Circles represent outliers.

Of biochemicals related to amino acid metabolism, the levels of tyramine, 2-methylbutyrylglycine, saccharopine, 5-aminovalerate, and methionine sulfoxide were increased 12.2-, 3.92, 2.40-, 2.15-, and 1.46-fold, respectively, in the intestinal contents of chicken fed the magnolia-supplemented diet, compared with unsupplemented controls (Figure 3). On the other hand, 3-(4-hydroxyphenyl)lactate, phenyllactate, 3-hydroxyisobutyrate, 2-hydroxy-3-methylvalerate, 5-oxoprolin, N2 N6-diacetyllysine, and N-acetylthreonine were measured at levels 0.15-, 0.21-, 0.23-, 0.38-, 0.38-, 0.46-, and 0.59-fold less than those in the unsupplemented controls (Figure 3). Of biochemicals related to lipid metabolism, 2-hydroxypalmitate, cholate sulfate, margarate, nervonate, and behenate levels were increased 5.46-, 4.79-, 4.43-, 3.08-, and 2.34-fold, respectively, in the magnolia-supplemented group, compared with unsupplemented controls, while glycerophosphoglycerol, glycerophosphoserine, and chiro-inositol were decreased 0.14-, 0.35-, and 0.45-fold, respectively (Figure 4A). Of biochemicals related to peptide metabolism, enterodiol levels were increased 9.09-fold, and those of erythritol, secoisolariciresinol, and naringenin were decreased 0.21-, 0.28-, and 0.39-fold in the magnolia *vs*. control groups (Figure 4B). Of biochemicals related to nucleoside metabolism, the levels of adenine, cytosine, 5-methylcystine, and orotidine were decreased 0.19-, 0.29-, 0.30-, and 0.36-fold in the magnolia *vs*. control groups (Figure 4C). Finally, erythronate, classified as carbohydrate metabolite, was decreased 0.56-fold, whereas 5-(2-hydroxyethyl)-4-methylthiazole, related to vitamin metabolism, was increased 31.1-fold in the intestinal contents of chicken fed the magnolia-supplemented diet, compared with unsupplemented controls (data not shown).

## Discussion

We previously reported that dietary supplementation of broiler chickens from 0 to 35 days of age with an extract of magnolia bark increased by 8.2% the average daily feed intake (79.3 to 85.8 g/chicken), by 10.6% the average body weight gains (50.9 to 56.3 g/chicken), and by 10.1% the final body weights (1828 to 2012 g/chicken), compared with chickens given an unsupplemented diet (12). Additionally, the prior study demonstrated that dietary magnolia supplementation improves antioxidant enzyme activities in the liver, spleen, and breast muscle of chickens, compared with unsupplemented controls. Pang et al. (15) reported that catalase and superoxide dismutase activities in plasma and liver increased following *in vivo* treatment of mice with magnolol and honokiol, the major active components of *M. officinalis*. Rajgopal et al. (16) showed that magnolia bark extract activated Nrf2-dependent heme oxygenase-1 gene expression *in vitro* in murine hepatocytes. Chang et al. (17) demonstrated an anti-atherogenic effect of *M. officinalis* associated with suppression of oxidative stress, measured by free radical, malondialdehyde, and oxidative DNA damage, and down-regulation of apoptosis-related gene expression, in hyperlipidemic rabbits. To the best of our knowledge, however, the current study is the first to characterize alterations in intestinal metabolite levels in poultry fed a magnolia-supplemented diet, compared with unsupplemented controls.

Total U.S. poultry meat consumption more than doubled from 15.5 g/person/day in 1960 to 42.5 g/person/day in 2018 (18). To meet this growing demand, U.S. broiler growth rates have doubled and corresponding feed conversion ratios (kg of feed per kg of body weight gain) have been reduced by approximately 50% over the past 60 years (19). Improvements in nutrition and genetic breeding stocks, and the introduction of AGPs, among others, have contributed to increasing the efficiency of commercial poultry production. In fact, most antibiotics are manufactured for use in food animal production. In the U.S., for example, total annual animal antibiotic usage is about 30 billion pounds, while total human use accounts for <10 billion pounds per year (20). AGP overuse in food animal production has led to the emergence of drug-resistant infectious pathogens that threaten human health (20, 21). As a substitute for AGPs, dietary supplementation with phytochemicals and micronutrients offers a potential alternative to increase chicken growth and decrease the appearance of drug-resistant bacteria (22–24). With the current experimental results, magnolia showed the potential as an antibiotic alternative and the intestinal biochemicals produced due to dietary magnolia supplementation may play a practical role in improving intestinal health, which may be linked to a growth-promoting effect in the chicken.

The bark of the magnolia plant, particularly *M. officinalis* and *M. obovata*, has been extensively used in traditional Chinese medicine for treating respiratory congestion, depression, and loss of appetite (8). Magnolol and honokiol activate both the peroxisome proliferator-activated receptor γ (PPARγ) homodimer and the PPARγ/retinoid X receptor heterodimer (25). PPARs comprise a family of transcription factors that bind to peroxisome proliferator hormone response elements in gene promoters, thereby regulating cellular differentiation, development, and metabolism (26). Synthetic and naturally-occurring PPAR agonists decrease triglyceride and blood glucose levels, and are used for treating metabolic disorders, including diabetes (27). Magnolol and honokiol are neolignans, two C_6_C_3_ units which are not β-β (8-8')-linked (28). Magnolol (4-allyl-2-(5-allyl-2-hydroxy-phenyl)phenol) exhibits a unique mechanism of binding to PPARγ (29). Two magnolol molecules occupy the PPARγ ligand-binding domain in a cooperative manner. One hydroxyl group of the first magnolol molecule forms a hydrogen bond with Ser-289 of PPARγ and hydrogen bonds with Tyr-473, while another hydroxyl group of the second magnolol molecule forms a hydrogen bond with Ser-342 with additional hydrogen bonding. Downstream pathways that have been identified following magnolol/honokiol binding to their cognate receptors include the NF-κB/MAPK, Nrf2/HO-1, and PI3K/Akt signaling cascades (30). Base on the aforementioned statement, the intestinal biochemicals in the current study might stimulate host intestinal epithelial and/or immune cells receptors. Future *in vitro* and/or *in vivo* studies of these or other receptors may be required to further understand a mechanism of these biochemicals in the intestine.

We observed that chickens given a diet containing *Bacillus subtilis*-based probiotics had a lower number of metabolic changes compared with the present report (31). More specifically, compared with unsupplemented controls, the levels of 83 metabolites were altered (25 increased, 58 decreased) in chickens given a diet supplemented with *B. subtilis* strain 1781, while 50 were altered (12 increased, 38 decreased) with a *B. subtilis* strain 747-supplemented diet. On the other hand, the metabolite level changes seen in the current study (165 increased, 113 decreased) are comparable to those we previously reported when analyzing intestinal metabolites in chickens fed a diet supplemented with the AGPs, virginiamycin and bacitracin (13). In the AGP study, the levels of 218 biochemicals were altered (156 increased, 62 decreased) in chickens given a virginiamycin-supplemented diet, while 119 were altered (96 increased, 23 decreased) with the bacitracin-supplemented diet, compared with unsupplemented controls. Similar to the current investigation, the most common metabolites altered by AGP supplementation included those of amino acids, fatty acids, nucleosides, and vitamins/cofactors. However, there was little overlap in the individual chemical compounds that were identified as the most dramatically increased or decreased between the two studies. For example, among the metabolites of amino acids most highly elevated in both the virginiamycin- and magnolia-supplemented diets were those related to lysine. Individually, however, while the levels of N6-formyllyisne, 5-hydroxylysine, and 2-aminoadipate were increased in the intestine of virginiamycin-supplemented chickens, the levels of saccharopine and 5-aminovalerate were most elevated in chickens given the magnolia-supplemented diet. A similar pattern was noted for the other chemical compounds identified in the two studies. These results may imply that intestinal metabolites have a more direct role of maintaining intestinal homeostasis than the intestinal microbiome because they are final products produced through the intestinal microflora. Therefore, whereas these combined results suggest a strategy that might be used to improve poultry growth performance without the use of in-feed antibiotics, additional studies are needed to identify specific metabolic alterations that might be exploited to increase chicken growth in the absence of AGP supplementation.

In summary, this report demonstrates that dietary supplementation with magnolia bark extract has profound effects on the levels of a wide variety of chemical metabolites in the chicken intestine, particularly those related to amino acids, fatty acids, peptides, and nucleosides. Compared with unsupplemented controls, these altered metabolite levels provide a biochemical signature unique to magnolia bark extract supplementation. Our result suggest that altered metabolites can be used to maintain intestinal homeostasis within epithelial or immune cells, which might account for their effects on overall gut health as well as chicken growth. Through future *in vitro* and/or *in vivo* studies, identification of the altered metabolites that confer properties of AGPs would suggest their potential use as antibiotic alternatives.

## Data Availability Statement

All datasets generated for this study are included in the article/supplementary material.

## Ethics Statement

The animal study was reviewed and approved by The Institutional Animal Care and Use Committee of the Beltsville Agriculture Research Center.

## Author Contributions

SO and HL designed the research. SO and HL conducted research. IP, EL, and HL analyzed data, wrote the paper and have responsibility for its content. All authors read and approved the final manuscript.

### Conflict of Interest

The authors declare that the research was conducted in the absence of any commercial or financial relationships that could be construed as a potential conflict of interest.

## References

[1] Ventola CL. The antibiotic resistance crisis: Part 1: causes and threats. PT. (2015) 40:277–83. 25859123 PMC4378521

[2] Ventola CL. The antibiotic resistance crisis: Part 2: management strategies and new agents. PT. (2015) 40:344–52. 25987823 PMC4422635

[3] Martin MJ Thottathil SE Newman TB. Antibiotics overuse in animal agriculture: a call to action for health care providers. Am J Public Health. (2015) 105:2409–10. 10.2105/AJPH.2015.302870 26469675 PMC4638249

[4] Moore PR Evenson A Luckey TD McCoy E Elvehjem CA Hart EB. Use of sulfasuxidine, streptothricin, and streptomycin in nutritional studies with the chick. J Biol Chem. (1946) 165:437–41. 20276107

[5] Gadde U Kim WH Oh ST Lillehoj HS. Alternatives to antibiotics for maximizing growth performance and feed efficiency in poultry: a review. Anim Health Res Rev. (2017) 18:26–45. 10.1017/S1466252316000207 28485263

[6] Lillehoj H Liu Y Calsamiglia S Fernandez-Miyakawa ME Chi F Cravens RL. Phytochemicals as antibiotic alternatives to promote growth and enhance host health. Vet Res. (2018) 49:76. 10.1186/s13567-018-0562-6 30060764 PMC6066919

[7] Grant A Gay CG Lillehoj HS. *Bacillus* spp. as direct-fed microbial antibiotic alternatives to enhance growth, immunity, and gut health in poultry. Avian Pathol. (2018) 47:339–51. 10.1080/03079457.2018.1464117 29635926

[8] Lee YJ Lee YM Lee CK Jung JK Han SB Hong JT. Therapeutic applications of compounds in the *Magnolia* family. Pharmacol Ther. (2011) 130:157–76. 10.1016/j.pharmthera.2011.01.010 21277893

[9] Prasad R Katiyar SK. Honokiol, an active compound of magnolia plant, inhibits growth, and progression of cancers of different organs. Adv Exp Med Biol. (2016) 928:245–65. 10.1007/978-3-319-41334-1_11 27671820

[10] Poivre M Duez P. Biological activity and toxicity of the Chinese herb *Magnolia officinalis* Rehder & E. Wilson (Houpo) and its constituents. J Zhejiang Univ Sci B. (2017) 18:194–214. 10.1631/jzus.B1600299 28271656 PMC5365644

[11] Ranaware AM Banik K Deshpande V Padmavathi G Roy NK Sethi G. Magnolol: A neolignan from the magnolia family for the prevention and treatment of cancer. Int J Mol Sci. (2018) 19:2362. 10.3390/ijms19082362 30103472 PMC6121321

[12] Oh S Gadde UD Bravo D Lillehoj EP Lillehoj HS. Growth-promoting and antioxidant effects of magnolia bark extract in chickens uninfected or co-infected with *Clostridium perfringens* and *Eimeria maxima* as an experimental model of necrotic enteritis. Curr Dev Nutr. (2018) 2:nzy009. 10.1093/cdn/nzy009 30019032 PMC6041942

[13] Gadde UD Oh S Lillehoj HS Lillehoj EP. Antibiotic growth promoters virginiamycin and bacitracin methylene disalicylate alter the chicken intestinal metabolome. Sci Rep. (2018) 8:3592. 10.1038/s41598-018-22004-6 29483631 PMC5827016

[14] Worley B Powers R. Multivariate analysis in metabolomics. Curr Metabolomics. (2013) 1:92–107. 10.2174/2213235X11301010092 26078916 PMC4465187

[15] Pang YL Han XF Bamikole MA Gong ZH Tang SX Tan ZL Anti-diarrhea and anti-oxidant properties of magnolol. Trop J Pharm Res. (2013) 12:85–91. 10.4314/tjpr.v12i1.14

[16] Rajgopal A Missler SR Scholten JD. *Magnolia officinalis* (Hou Po) bark extract stimulates the Nrf2-pathway in hepatocytes and protects against oxidative stress. J Ethnopharmacol. (2016) 193:657–62. 10.1016/j.jep.2016.10.016 27721050

[17] Chang WC Yu YM Hsu YM Wu CH Yin PL Chiang SY. Inhibitory effect of *Magnolia officinalis* and lovastatin on aortic oxidative stress and apoptosis in hyperlipidemic rabbits. J Cardiovasc Pharmacol. (2006) 47:463–8. 10.1097/01.fjc.0000211708.03111.6e 16633091

[18] Per Capita Consumption of Poultry and Livestock 1960 to Forecast 2020 in Pounds National Chicken Council. (2019). Available online at: https://www.nationalchickencouncil.org/about-the-industry/statistics/per-capita-consumption-of-poultry-and-livestock-1965-to-estimated-2012-in-pounds/ (accessed March 13, 2020).

[19] Thiruvenkadan A Prabakaran R Panneerselvam S Broiler breeding strategies over the decades: An overview. World's Poult Sci J. (2011) 67:309–36. 10.1017/S0043933911000328

[20] Marshall BM Levy SB. Food animals and antimicrobials: impacts on human health. Clin Microbiol Rev. (2011) 24:718–33. 10.1128/CMR.00002-11 21976606 PMC3194830

[21] Kumar S Singh BR An overview of mechanisms and emergence of antimicrobial drug resistance. Adv Anim Vet Sci. (2013) 1:7–14.

[22] Ademola IO Ojo PO Odeniran PO. *Pleurotus ostreatus* extract inhibits *Eimeria* species development in naturally infected broiler chickens. Trop Anim Health Pro. (2019) 51:109–17. 10.1007/s11250-018-1665-9 30008132

[23] Wunderlich F Al-Quraishy S Steinbrenner H Sies H Dkhil MA. Towards identifying novel anti-*Eimeria* agents: Trace elements, vitamins, and plant-based natural products. Parasitol Res. (2014) 113:3547–56. 10.1007/s00436-014-4101-8 25185667

[24] Quiroz-Castañeda RE Dantán-González E. Control of avian coccidiosis: Future and present natural alternatives. Biomed Res Int. (2015) 2015:430610. 10.1155/2015/430610 25785269 PMC4346696

[25] Wang L Waltenberger B Pferschy-Wenzig EM Blunder M Liu X Malainer C. Natural product agonists of peroxisome proliferator-activated receptor gamma (PPARγ): a review. Biochem Pharmacol. (2014) 92:73–89. 10.1016/j.bcp.2014.07.018 25083916 PMC4212005

[26] Michalik L Auwerx J Berger JP Chatterjee VK Glass CK Gonzalez FJ. International Union of Pharmacology. LXI. Peroxisome proliferator-activated receptors. Pharmacol Rev. (2006) 58:726–41. 10.1124/pr.58.4.5 17132851

[27] Staels B Fruchart JC. Therapeutic roles of peroxisome proliferator-activated receptor agonists. Diabetes. (2005) 54:2460–70. 10.2337/diabetes.54.8.2460 16046315

[28] Gottlieb OR. The rational search for natural neolignans. Mem Inst Oswaldo Cruz. (1991) 86(Suppl. 2):25–9. 10.1590/s0074-02761991000600009 1668770

[29] Zhang H Xu X Chen L Chen J Hu L Jiang H. Molecular determinants of magnolol targeting both RXRα and PPARγ. PLoS ONE. (2011) 6:e28253. 10.1371/journal.pone.0028253 22140563 PMC3226690

[30] Zhang J Chen Z Huang X Shi W Zhang R Chen M. Insights on the multifunctional activities of magnolol. Biomed Res Int. (2019) 2019:1847130. 10.1155/2019/1847130 31240205 PMC6556366

[31] Park I Zimmerman NP Smith AH Rehberger TG Lillehoj EP Lillehoj HS. Dietary supplementation with *Bacillus subtilis* direct-fed microbials alters chicken intestinal metabolite levels. Front Vet Sci. (2020) 7:123. 10.3389/fvets.2020.00123 32195276 PMC7064633

